# The Truth is in the Data – Differences in the Same Measure Based on Different Sources among HVHC Members Using ICU Length of Stay as an Example

**DOI:** 10.5334/egems.194

**Published:** 2017-12-15

**Authors:** Friedrich Maximilian von Recklinghausen, Andreas Taenzer, Chrissie Gorman, Jay Knowlton, Allison Kinslow, Ron Russell

**Affiliations:** 1 Dartmouth College, US; 2 Geisel Medical School at Dartmouth, US; 3 High Value Healthcare Collaborative, US

**Keywords:** data, CMS, electronic health record, EHR, sepsis, ICU

## Abstract

**Introduction:**

Intensive Care Unit (ICU) length of stay is a strong indicator of severity of illness and cost in the care of sepsis patients. In this case study, we examine the difference between an electronic health record (EHR) based submissions with Centers for Medicare and Medicaid Services (CMS) payment data.

**Methods:**

Member submitted EHR data contained 26,733 unique patient’s records. The CMS data contained demographics, diagnosis, and revenue codes. After linking EHR data to CMS data, we found a discrepancy in ICU days from CMS claims vs. EHR data. Our hypothesis was that removing intermediate ICU LOS would result in a closer match from CMS claims with EHR data. We suspected the use of Intermediate ICU stays in our CMS ICU definition contaminated our ICU LOS data. This resulted in a review of the sepsis specification, further investigation of the data, and follow up conversations with the Member organizations.

**Results:**

Agreement between EHR and CMS data improved from 73 percent to 86 percent once the Intermediate ICU time had been removed.

**Discussion and Conclusions:**

The inclusion of Intermediate ICU in the analysis of severely ill sepsis patients from CMS data diluted the importance of using an ICU LOS for estimating the severity of illness and the cost to the healthcare system. We must ensure that clinical definitions are consistent between data sources that were built for different purposes. Additionally, we learned that engaging with clinicians, analysts, and clinical coders early in the process is required to fully understand the complexities from different sources.

## Introduction

Intensive Care Unit (ICU) length of stay is a strong indicator of severity of illness, resource usage, and cost in the care of sepsis patients. As such, it is important to accurately measure the ICU length of stay (LOS) [1]. However, there are several different types of data used in the health care setting (e.g., clinical, billing, research, and quality improvement); each with a different source and purpose. It is quite possible to measure the same thing from different sources and get different values. In this case study, we review the processing of two of these types of data, the resultant discrepancy, and an acceptable resolution. In particular, we examine the difference between a specification of electronic health record (EHR) based submissions of ICU LOS and the same measure based on Centers for Medicare and Medicaid Services (CMS) payment data. This is important for benchmarking and reporting, payment, national policy, and quality improvement and research endeavors.

The High Value Healthcare Collaborative (HVHC) is a provider learning network of health care systems across the country committed to improving health care value through data and collaboration. Our goals are to:

- Measure, innovate, test, and continuously improve value-based care.
- Rapidly disseminate and facilitate adoption of proven high value care models across HVHC Members and beyond.
- Advocate for policy and payment models that support sustainable high value health care [2].

Two groups from HVHC were responsible for the creation of a sepsis data specification. This specification defined the data elements to be collected from EHR systems as well as the structure and format of the data. The first group was the Sepsis Dissemination and Implementation (D&I) Project Team. This group was comprised of physicians, health services researchers, population health experts, data scientists, and Program Management Office (PMO) staff (for facilitation and coordination). The second group was the HVHC Data Stewards subcommittee. Data Stewards, in conjunction with other clinical teams, are responsible for the design of data extraction specifications for Members. They also guide the initial semi-automated quality control (QC) process referenced below.

During 2016 and 2017, HVHC and its PMO received submissions of sepsis care data from Member organizations. These data were extracted from EHR or other clinical systems based on the data specification, with ICU LOS generally extracted from the clinical Admission, Discharge, and Transfer (ADT) systems [3]. Prior to any analysis, the data went through a significant quality control process that identified misaligned or bad data, as described in Knowlton, et al. in this issue. Once these data had been processed, they were linked to CMS patient data using direct patient identifiers (e.g., social security number) contained within in the Member submission; these patient identifiers were submitted and stored in a separate and secure location to maintain compliance with Federal privacy rules, Institutional Review Board mandates, and the HVHC Master Collaboration Agreement (MCA).

In contrast, identification of ICU LOS from the CMS claims data was based upon a definition provided by RESDAC:

Intensive Care Day Count, derivation, this field is derived by accumulating the revenue center unit count associated with accommodation revenue center codes 020X (all 9 subcategories) from all claims included in the stay [4].

## Methods

Member-submitted EHR data contained 26,733 unique patients’ records including both administrative components (such as demographics) and clinical components (such as medications and vital signs). The CMS data contained demographics, diagnosis, and revenue codes. Both data sources contained the elements required to calculate ICU LOS.

Matching the Member EHR data to the CMS claims data required a set of patient-level identifiers that were used to generate a likelihood of a true match. These identifiers included patient code, health insurance claim number (HICN), social security number (SSN), date of birth, gender, first name, last name, and ZIP code. Identifiers were ranked based on their ability to generate accurate matches (see Table 1). These various combinations were necessary to account for variability in available data across Members. The matching resulted in 2,396 (8 percent) of the Member organizations’ sepsis encounters (that presented in the Emergency Department) being found in the CMS data. These 2,396 cases accounted for 45 percent of the total number of cases with a potential Medicare match. Reasons for the non-matching records include age restrictions (less than 65 or greater than 99), HMO eligibility (data restricted to FFS claims), dual eligibility with Medicaid, and sepsis encounters with zero (or missing) cost.

**Table 1:** Primary keys for identifying patients.

Order of Preference	PHI Fields Required	Comments

1^st^ preference	**PAT_CODE, HICN, DATEOFBIRTH, SEX, ZIPCODE**	**PAT_CODE** is a unique member-defined code that should not change from submission to submission for the same patient and should always be present. **HICN** is the unique Medicare ID value used to link member submitted data to CMS administrative data. **DATEOFBIRTH, SEX, ZIPCODE** are always required as they are used in the analytic processing of the data to support outcomes reporting, they are also useful fields in validating linkage work.
2^nd^ preference	**PAT_CODE, SSN, DATEOFBIRTH, SEX, ZIPCODE**	**PAT_CODE** is a unique member-defined code that should not change from submission to submission for the same patient and should always be present. **SSN** is the unique patient SSN value used to link member submitted data to CMS administrative data and commercial administrative data (if available). **DATEOFBIRTH, SEX, ZIPCODE** are always required as they are used in the analytic processing of the data to support outcomes reporting, they are also useful fields in validating linkage work.
3^rd^ preference	**PAT_CODE, FIRSTNAME, LASTNAME, DATEOFBIRTH, SEX, ZIPCODE**	**PAT_CODE** is a unique member-defined code that should not change from submission to submission for the same patient and should always be present. **FIRSTNAME, LASTNAME** are the unique patient name values used to link member submitted data to CMS administrative data and commercial administrative data (if available). This linkage method is not exact and can lead to false positives. **DATEOFBIRTH, SEX, ZIPCODE** are always required as they are used in the analytic processing of the data to support outcomes reporting, they will also be used for linkage to CMS or commercial administrative data if only the first and last name values are submitted.
Other	Any one of the three above with **MRN** also added	**MRN** is the member hospital unique patient Medical Record Number. This data element is not required for linkage to any administrative data, but is used to link a patient’s records together if a patient’s PAT_CODE was to change from one data submission to another. This field can be included in any of the preference combinations listed above.

After linking the Member organizations’ EHR data to the CMS claims data, we found a discrepancy when comparing ICU days sourced via CMS claims vs. Member-submitted data for CMS-linked sepsis encounters. Of those records that matched, there were 483 (20 percent) encounters with zero ICU LOS from the Member data, but non-zero ICU LOS from the matching CMS data. This resulted in a review of the sepsis specification [4], further investigation of the data, and follow-up conversations with the Member organizations.

Our initial review focused on a cross-tabulation of patient encounters with and without ICU LOS in the matched data. As shown in Table 2, this analysis revealed a discrepancy between data sources. There were 483 (20 percent) sepsis cases where the EHR contained zero ICU LOS, but the matching CMS claim had a non-zero ICU LOS. This indicated two possibilities: either the Members had not submitted some ICU LOS encounters to CMS, or we were over counting in the CMS data. Either way, some Member organizations were submitting far fewer ICU LOS days than were indicated by the CMS data.

**Table 2:** Patient encounters with ICU LOS.

		Member-Submitted Data		
		NO ICU LOS	HAS ICU LOS	TOTAL

CMS Claims Data	NO ICU LOS	737 (31%)	170 (7%)	907
	HAS ICU LOS	483 (20%)	1,006 (42%)	1,489
	TOTAL	1,220	1,176	2,396

Following the initial analysis, we undertook a review of the EHR sepsis specification [5], frequently asked questions (FAQ) document, and meeting minutes with the Data Stewards. From this review, we were confident that the ICU LOS from the Member-submitted EHR data was based upon patients that occupied an ICU bed and needed ICU services. The ICU LOS language in the data specification, Table 3, is very clear. The review of the FAQ document and meeting minutes both confirmed that the ICU patients should fulfill both criteria of occupying an ICU bed and receiving ICU services.

**Table 3:** ICU LOS definition from Sepsis specification (4).

Tag Value	Required	Tag Definition	Valid Code Values

DISSEP401	Y, only if patient in ICU	ICU length of stay for sepsis event	Integer: Length of stay in days, rounded to nearest integer

The next step we took was to contact the data staff from the Member organizations. We reached out to individuals from the HVHC Data Stewards as they were well engaged in the process. Discussions with a sample of five of the Member organizations’ data representatives were enlightening. We were assured that the submissions adhered to the definition of ICU bed (for location) and ICU requiring services (for level of care) for the submitted encounters. As a part of these discussions, we asked Member organizations whether the source of the total LOS was administrative or clinical data. This review included the ICU, general care or floor beds and a level of care in between. The care received between ICU and a general bed has various names across Member organizations, such as step down, progressive care, etc.

We suspected that the problem might be due to the use of Intermediate ICU stays contaminating our ICU LOS data pulled from CMS data, thus we set out to investigate further. Our hypothesis was that removing ICU LOS sourced via Revenue Center 0206 (intermediate ICU) would result in a closer match in comparing ICU LOS from CMS claims with Member-submitted data.

The concept of the intermediate ICU evolved over the last 30 years and represents a small portion of the overall time that can be billed as an ICU [5]. As the usage of the Intermediate ICU was ill defined, a clarification from CMS was promulgated:

There is approximately a 20% error rate in the revenue center code category 0206 due to coders misunderstanding the term ‘post ICU’ as including any day after an ICU stay rather than just days in a step-down/lower case version of an ICU. ‘Post’ was removed from the revenue center code 0206 description, effective 10/1/96 (12/96 MEDPAR update). 0206 is now defined as ‘intermediate ICU’ [4].

All ICU revenue codes are listed in Table 4. Intermediate ICU CMS billing accounted for 1,046,936 hospitalizations in 2010 as compared with 553,600 for the ICU and 2,033,360 for general or ward care [3]. The use of the Intermediate ICU increased over the period 1996 to 2010 [3].

**Table 4:** ICU Revenue Codes.

Code	Type

0200	General
0201	Surgical
0202	Medical
0203	Pediatric
0204	Psychiatric
0206	Intermediate ICU prior to 12/96 update was ‘post ICU’
0207	Burn care
0208	Trauma
0209	Other intensive care

The EHR sepsis specification [5] was intended to describe ICU LOS as the physical location of the patient and the level of care the patient was receiving. For example, a patient occupying a bed in the ICU who does not need ICU level of care would not contribute to the ICU LOS, while a patient occupying a bed requiring ICU level of care would contribute to the ICU LOS.

An analysis of the CMS data began based upon the review of the specification, and initial review of the data and discussions with the Member organizations. The first steps involved a deep review of the ICU revenue codes. We found multiple encounters with the 0206 intermediate ICU code being attributed to the CMS-sourced ICU LOS. We removed revenue code 0206 from the CMS ICU definition and recalculated ICU LOS comparisons.

## Results

Table 5 displays the cross-tabulation of encounters after the intermediate ICU LOS was excluded from the CMS data. Our goal was to improve the alignment of the ICU assignment between the data sources. Overall, removing code 0206 from the ICU definition reduced the number of encounters with time in the ICU from 1,489 to 1,080 (–27 percent). These 409 encounters would have only received intermediate ICU care. The number of encounters where both data sources agreed on the presence of ICU time (top left and bottom right cells) increased from 1,743 to 2,072 (19 percent). The number of misaligned encounters (top right and bottom left cells) decreased from 653 to 324 (–50 percent). The net effect across all encounter was to improve alignment from 73 percent to 86 percent.

**Table 5:** Patient encounters with ICU LOS with Intermediate ICU removed.

		Member-Submitted Data		
		NO ICU LOS	HAS ICU LOS	TOTAL

CMS Claims Data	NO ICU LOS	1,113 (46%)	203 (8%)	1,316
	HAS ICU LOS	121 (5%)	959 (40%)	1,080
	TOTAL	1,234	1,162	2,396

## Discussion and Conclusions

In this study, we describe and analyze the effect of including the intermediate ICU LOS when matching Member-submitted EHR data to CMS data. Removing ICU LOS sourced via revenue center code 0206 (intermediate ICU) would result in a closer match between ICU LOS from CMS claims and Member organization-submitted EHR data. This resulted in a greater agreement of 329 sepsis encounters linked from Member-submitted data to CMS claims.

The inclusion of Intermediate ICU in the analysis of severely ill sepsis patients from CMS data diluted the importance of using an ICU LOS as a metric for estimating the severity of illness and the cost to the health care system of sepsis patients.

While removal of the intermediate ICU code improved agreement between the data sources, it did not resolve the differences entirely. The reason for the remaining discrepancy is unclear. We speculate that some of difference may be due to the discretion of clinicians versus coding systems in identifying ICU time. For example, ICU time submitted by Members was drawn directly from medical records with time stamps accompanying the notes. In contrast, ICU time from the CMS claims was generated through billing systems that follow coding algorithms and use daily sweeps of the patient’s status.

There are potential impacts from this study. It changed our understanding of both Member organization EHR data and CMS claims data and reinforced that we have to be very careful to ensure that clinical definitions are consistent between data sources that were built for different purposes [6]. A robust QC process early in the analysis is critical for identifying cases for correction or resubmission before producing final analytics. We learned that engaging with clinicians, analysts, and clinical coders together is required to fully understand the complexities of EHR data and how it may differ from administrative claims. Having those discussions early in the process helps to ensure that outcomes are relevant to clinicians, the audience of interest for this purpose.

Our findings have impact on our future research. Having a clear set of definitions and data sets with high agreement allow for benchmarking, both for the individual Member organizations and for HVHC. We had a much better understanding of the reimbursement from CMS and how the inclusion of Intermediate ICU would affect it. This study has given us some future areas of research, including reimbursement for different health conditions, alignment of differing data sets, and quality improvement at the local level.

The disparity between billing and clinical data suggests the need for health services researchers to have a deep understanding of data to be fit for use; for example, many studies leverage claims data for analysis, which has been shown to have a notable disconnect from clinical care [7]. It further clarified our use of the ICU LOS metric as an indicator of both illness severity and cost in sepsis patients. It illustrated that definitions are crucial when combining datasets from different sources. It used teams of physicians, analysts, and coordinators to compile this study.

This study has several limitations. This was a limited, small dataset, further decreased in size by linking only patients over 65 on fee for service Medicare to CMS records. It was also restricted to only patients with severe sepsis or septic shock from specific health care organizations. It may not be generalizable to the entire population.
